# Role, Development, and Value of Enzymatic Debridement as Integral Component in Initial Treatment of Burn Injuries Exemplified by NexoBrid^®^

**DOI:** 10.3390/ebj3020029

**Published:** 2022-04-21

**Authors:** Maximilian M. Mattern, Paul C. Fuchs, Jennifer L. Schiefer

**Affiliations:** Department of Plastic Surgery, Hand Surgery and Burn Center, Cologne-Merheim Medical Center (CMMC), University of Witten/Herdecke, Ostmerheimer Str. 200, 51109 Cologne, Germany; fuchsp@kliniken-koeln.de (P.C.F.); schieferj@kliniken-koeln.de (J.L.S.)

**Keywords:** burn injury, eschar, enzymatic debridement, bromelain, NexoBrid^®^, burn center

## Abstract

Despite intensive research and increased knowledge over the past decades, the handling of severe burn injuries remains complex and is mainly based on clinical experience. High demands in terms of the diagnosis and choice of therapy often confront clinicians with challenging circumstances. Thus, the treatment of burn injuries has predominantly remained under the responsibility of specialised centres. As a new approach in addition to conventional surgery, enzymatically controlled debridement has come into focus for the treatment of burn injuries over the past years. The efficacy and safety of enzymatic debridement has already been implemented by numerous reputable studies. Promising results from the literature are enhanced by feedback from various conference contributions, intradisciplinary exchanges, and international collaborations. The implementation of enzymatic debridement in initial care management was found to be capable of reforming Standards of Care in numerous burn centres by facilitating treatment determinations and reducing the number of classical surgical interventions. Nevertheless, its use is also subject to certain restrictions as usage has shown limitations concerning efficacy when applied to scalds or pre-treated wounds. Enzymatic debridement shows high efficacy in terms of tissue debridement by combining this feature with the minimisation of collateral damage and a broad field of application in burn injuries. Due to their impressive performance in the treatment of burn injuries, enzyme-based techniques have also attracted attention for the treatment of other pathologies such as chronic wounds and are objects of ongoing research in this field. In this article, we illustrate the significance of enzyme-based treatment in initial burn care and shed some light on the potential value of enzymatic approaches in future burn surgery.

## 1. Introduction

Burn injuries of miscellaneous forms represent a common clinical picture in emergency rooms around the world and, thus, often confront inexperienced clinicians with demanding challenges. The relatively high prevalence of burn injuries can be explained by their manifold aetiology, including scalds, burns, frostbite, and radiation, electrical, or chemical accidents, comprising a large cohort of exposed and vulnerable people.

Despite their reported high prevalence of 11 million global incidents per year according to statistics of the World Health Organisation (WHO), data on burn injuries are still poor due to the significant underrepresentation of low developed countries [1,2,3]. In this context, the WHO has already started to establish an international online registry aiming for standardized reporting of burn injuries [4]. The need of centralized and more comprehensive registration for better representation of lower developed countries is further manifested when also taking cultural differences into account, such as popular cooking over open fire [5,6,7]. In more developed countries with structured statistical recording, such as that conducted by the American Burn Association (ABA), data analysis reveals twice as many injured males compared to females, with peaking incidences in young children (around 1–16 years-old) and adults of working age (20–59 years old) [8]. Interestingly, available data from mid- or lower-developed countries report a reversed gender distribution with more women affected, which may at least partly be explainable with higher persistence of traditional distribution of gender roles or/and domestic violence compared to developed countries [9,10]. Among the aforementioned aetiologies, the ABA refers to classic flame burns as the most common mechanism of injury (41%) in the US, followed by scalds (31%), while chemical (3.5%) or electrical injuries (3.6%) tend to be less frequent [8]. However, the distribution may differ in more vulnerable populations. Expectedly, in children below an age of five years, scalds represent the dominant cause for burn injuries, whereas prevalence in people with predisposing illnesses such as epilepsy is reported to be increased [11,12].

Observed tissue damage originates from thermal energy transfer and predominantly affects dermal and subcutaneous structures, but it is also capable of leading to severe damage of deeper structures such as the fascia, muscles, or bones. The extent and severity of tissue damage are mainly based on both temperature and exposure time but may additionally be influenced by the mechanism of trauma and their individual characteristics such as deepening in scalds or hidden involvement of deeper tissue layers in electrical accidents. The first evaluation of total body surface area (TBSA) for general overview is often performed by using the Wallace rule of nines, which is partitioning the adult human body into sectors of 9% or 18%, respectively [13]. More precise and common for spotty and irregular circumscribed burns is the so-called “rule of palm”, predicating congruence of the patient’s palm (incl. fingers) to 1% body surface area (BSA). Precise examination of depth extension is mandatory for proper assessment of severity, adequate volume management, and correct determination of further therapy, consisting of a multilayered surgical or conservative management.

Special attention is required in case of circumferential burns when affecting both the trunk and limbs. Circumferential burns with minimized skin elasticity pose a risk for the development of burn-induced compartment syndrome (BICS), which can lead to severe ventilation issues and high intra-abdominal pressures in case of thoraco-abdominal burns or to serious, irreversible tissue destruction when affecting the extremities [14]. For prophylaxis as well as treatment, surgical escharotomy represents the first choice [15]. 

Depending on the affected tissue layers, injuries can be classified as superficial-, partial-, or full-thickness burns: Whilst superficial or first-degree burns only mildly affect the epidermis and result in time-limited, painful erythema, partial thickness burns can be further divided into superficial partial- (also known as 2A burns) and deep partial-thickness burns (also known as 2B burns), depending on the involvement of only papillary or also reticular dermis. Whereas superficial partial burns go along with strong pain and blistering but sufficient spontaneous healing prospects, deep partial-thickness burns may also show blisters but only attenuated nociception, a faint wound bed, and high risk of scarring and prolonged healing. On the contrary, full thickness burns (third-degree burns) reach to deep dermal and subdermal structures but may even extend to muscles or bones (in literature often reported as fourth-degree burns) and commonly present dry, insensitive, and with a leathery fell of the skin. Spontaneous healing prospects are nearly abolished in this state, generally leading to an obligatory indication for surgical intervention and skin grafting.

However, challenges regarding the correct valuation of oftentimes heterogenous wound patterns are part of ongoing research and have already led to the use and, partially, the implementation of novel but expensive and rare technologies such as laser Doppler imaging (LDI), ultrasound, and several other technologies in order to counter this issue [16,17,18]. Due to the fundamental importance of the correct evaluation of intricate burn patterns for appropriate treatment, the complex underlying pathophysiology, and potentially severe systemic responses, the clinical handling of patients with thermal injuries remains highly demanding [19]. Depending on the extent of injury, cardiocirculatory conditions, the presence of combined injuries, or suspected chemical or physical inhalation trauma with potential respiratory dysfunction, patients often require monitoring or intensive care. 

For these reasons, assessment, diagnostics, and therapy are widely incumbent upon highly specialized centres and experienced physicians in order to provide best medical care and outcome. For the facilitation of decision-making, strict indications for the transfer of burnt patients to specialized centres are clearly defined in order to guarantee a suitable treatment of complex cases on one hand and to prevent overloading of burn institutions on the other hand (see Table 1; from Guideline of the German Society for Burn Medicine (DGV), February 2021). Nevertheless, peripheral clinics frequently remain the initial entry point of sometimes even heavily burnt patients before relocating to appropriate centres.

## 2. Enzymatic Debridement

Still, also after the transferal of patients to specialized burn centres, the involvement of complex anatomical areas such as the hands, face, or genital region often also puts experienced specialists in challenging situations [20,21,22]. The limited soft tissue sheath and, consequently, superficial exposition of sensitive functional structures set high demands on classical surgical debridement, which to date still represents the gold standard treatment. For this reason, the development and application of more considerate techniques, first and foremost, the enzymatic debridement (ED), has come into focus of today’s burn surgery [23,24]. The early removal of necrotic eschar represents the standard in modern burn therapy in order to reduce or prevent local infection, sepsis, scarring, or other complications and allow proper wound bed evaluation. Enzymatic debridement offers a needs-based technique with a maximum of healthy tissue conservation by providing a selective and sparing concept for necrotic tissue ablation [25,26,27]. The application of plant-based agents such as resin, honey, or other herbal remedies on burn wounds was already described in ancient Egypt (1600 BC) [28]. However, it has taken until the middle of the last century for substances such as papain, debricin, and other plant-derived enzymes to be applied in burn surgery, but despite their rapid and sufficient results, they were never implemented due to laborious production, poor quality, and lack of standardization [29,30,31,32,33]. In the 1970s, Levine et al. observed promising results with good debriding effects in animal tests by using a pineapple-derived enzyme mix, called “Bromelain” [34,35]. Still, the analysis of the exact chemical composition as well as standardized manufacturing remained major barriers for the establishment of bromelain-derived approaches in the wide market [36]. With current technologies, these impediments were easily overcome. To date, studies also focus on histological changes before and after enzymatic treatment for a further understanding of the underlying pathophysiological effect mechanism. After use of ED, zones of necrosis, stasis, or hyperemia reveal a nearly complete wipe out of necrotic tissue, significant vascular congestion, and an expansion of inflammatory cells, whereas the surrounding unaffected dermis is completely spared. Furthermore, histological preparation uncovers the formation of a new layer in the upper area of dermal remnants, which is frequently described as “homogenized” and only shows a few remaining viable epithelial elements [37,38]. However, under ideal conditions and protection from desiccation, this layer is discussed to be the origin of the observed spontaneous re-epithelization capabilities after application of enzymatic debridement and renunciation of skin grafting [37].

As a bromelain-based substance named NexoBrid^®^ (NXB), distributed by MediWound GmbH (Rüsselsheim, Germany), represents the predominantly used product in Western burn centres and, thus, has now been widely established in initial burn treatment, the information on ED in this review largely focuses on the specifications of NXB. However, various considerations may be generally applicable for enzymatic debridement in burn injuries as advantages and problems may be partly, but not always, dependent on the efficient ingredient. Since the approval of a bromelain-based enzyme mix NexoBrid^®^ by the European Medication Association (EMA) for treatment of certain burn patterns in 2013, enzymatic treatment has become an essential part of the initial management of burn injuries. Still—as already addressed above—correct evaluation of burn depth plays a major role in initial burn assessment due to its crucial importance for the determination of correct therapy, including ED treatment [39,40]. For this reason, in the field of burn surgery, there have been several attempts over the years to shift wound and wound bed evaluations from primarily subjective and experience-based estimations to more objective techniques with consequently higher interrater reliability. For this purpose, different technologies have found their way into primary care of burn injuries. Whereas clinical evaluation based on sensation or appearance still represents the widely set standard, this technique has been supplemented by various devices in the meantime [41,42,43,44,45]. Whereas appliances such as nuclear magnetic resonance [46], laser speckle imaging [47], ultrasonography [48], or thermography [49,50] have never reached wide clinical acceptance, other techniques such as laser doppler flowmetry or laser doppler imaging (LDI) were able to establish themselves in burn depth assessment [39,51,52]. LDI is used for the measurement of burn wound perfusion, quantifying and thereby objectifying the visually gauged capillary refill time in order to distinguish between more superficial or deeper burn wounds in the context of healing tendencies. Expectedly, studies hypothesized that wounds with higher wound bed perfusion in LDI show better spontaneous healing capabilities [51]. Compared to clinical wound evaluation, which achieves a reported accuracy of 50–75% also when performed by experienced clinicians [41,53,54,55,56,57,58]. LDI has proven to be superior by several studies and meta-analyses, achieving values around 89–91% for sensitivity and 93–96% specificity [59,60]. Despite LDI’s undisputed contribution to a higher diagnostic accuracy, a recent survey in 2021 by Claes et al. among international burn centres unveiled the application of LDI at only 52% for at least 10 years [52]. Major obstacles for the implementation of LDI seem to be mainly of economic reasons due to high acquisition costs and inadequate reimbursement by health systems [39]. Due to its long period of application for over 20 years, LDI is considered as a firmly established and the most evidence-based method for objective burn depth assessment [52]. Nevertheless, results of this technique are also dependent on several factors which may distort LDI-reliability: Incomplete debridement with residual debris pretends deeper burn patterns, which may result in misjudgment and inappropriate treatment determination. Additionally, also the angle of the LDI-probe, motion artefacts, or previously applied silver coated wound dressing have been shown to interfere with the obtained results [61]. However, the existence of already satisfactory techniques has not brought the development of new approaches to standstill, still aiming for higher convenience, accuracy, or cost efficiency. Technologies such as in vivo microscopy by confocal-laser scanning for differentiation between superficial partial- and deep partial-burns on a histomorphological level obtain encouraging results but have not found widespread application so far [62].

After clinical and/or technological determination of the injury pattern, the suitability of the patient for inclusion to the ED treatment pathway should be evaluated in consensus with European admission criteria for NXB [63]: Official market approval comprises use in deep partial- to full-thickness burns, as long as the treated body surface area (BSA) does not exceed 15% due to a lack of data regarding application to larger areas. In terms of contraindication, NXB may officially not be applied in children or in case of hypersensitivity to its ingredients (bromelain, papain). Particular caution is advised for cases with co-occurrence of compromised cardiopulmonary conditions (e.g., IHT, COPD, CHD). Additionally, it is recalled that anew exposition towards bromelain-containing substances may end in an allergic response and that there is so far no recommendation for use on chemical, radiation-induced, or penetrating burn wounds. However, the modalities of NXB-application have been under continuous revaluation by leading European experts over the last years, leading to largely expanded consensus-based recommendations beyond previously described official marketing authorization [64,65].

## 3. Application Process

The next step after the confirmation of patient suitability and presence of deep partial- to full-thickness burns is the choice of an adequate analgesia regime as enzymatic debridement represents a painful procedure. Due to the selective eschar removal with conservation of non-affected structures, NXB is primarily used when the preservation of healthy tissue is a major concern. Therefore, enzymatic debridement has frequently been performed on limbs or facial burns [66,67,68,69].

### 3.1. Anaesthesia

In this context, the treatment of extremities is predestined for the deployment of regional anaesthesia procedures. This practice has already been proven to provide sufficient analgesia for enzymatic therapies by several studies [70,71,72]. In case of disseminated or larger burn patterns intravenous sedation and analgesia with mechanical ventilation is often applied as standard care [72]. However, certain burn patterns, as well as demarcated facial burns, have also been described to be painlessly treated via enzymatic debridement in analgosedation without ventilation or even local anaesthesia, establishing further methods for sufficient pain management [67].

### 3.2. Preliminary Work

Following the guidelines of the manufacturer [73] or handling instructions of the Federal Institute for Pharmaceuticals and Medical Products (BfArM) [74], respectively, it is necessary to properly clean wounds prior to NXB application for removal of all coarse impurities, soot, blisters, or previously applied ointments, which may attenuate the debriding capabilities. It is indicated that no silver or iodine compounds should be used when processing NXB, as Schulz et al. have uncovered the occurrence of interactions between NXB and various agents leading to an impairment of the debriding effects [75]. Internally, a polyhexanide solution is ordinarily used when performing enzymatic debridement. After cleaning, using common methods such as rubbing with sterile gauze, soaking in saline solution, and cleaning with a sponge, antibacterial-solution-soaked wound compresses are applied to the cleaned burns and left for at least two hours. This period serves to prevent wound bed from microbial contamination and desiccation—which is to be avoided especially in order to inhibit formation of neo-eschar [37]—and is called the pre-soaking phase. This step can be expanded to a significantly longer period, for example, due to resource management; however, compresses have to remain moist and need to be changed at least every 12 h when the completion of enzymatic debridement is postponed. Outside official admission, application of NXB without pre-soaking can be considered, e.g., for resource management reasons or prioritized start of treatment. This “fast-track-application” is commonly performed at the author´s clinic in emergency treatment with equivalent results and may especially become important in case of BICS prophylaxis.

### 3.3. Preparation of NXB

Before the mixing of NXB components can start, the examiner needs to estimate the size of the treatment area as Nexobrid^®^ is distributed in two sizes: 2 g, which is sufficient for 100 cm^2^ (≈1% BSA), or 5 g, which is sufficient for 250 cm^2^ (≈2.5% BSA), according to the manufacturer. Afterwards, the appropriate amount of NXB powder is mixed with the included gel (ratio 1:10) by using a sterile spatula until the formation of a homogeneous, light brown mixture. This mixture has to be applied to the treatment area within 15 min. The layer thickness should be around 1.5–3 mm. Prior to application, it is recommended to separate and protect surrounding unaffected tissue as well as vulnerable structures such as orifices, mucosae, or penetrating wounds, e.g., after surgical escharotomy, by surrounding the treated area with fatty ointment. In practice, fat gauze has been shown to be equally sufficient.

Following, the treated wound should be covered with sterile occlusive film dressing, which may adhere to the NXB gel-mix, as well as to the adhesive fat barrier. Any air remaining under the film dressing should be avoided to prevent gross dislocation of the applied paste. Dislocation can additionally be obviated by applying a loose and puffy dressing, which can be further fixated via stabilizing gauze bandages.

### 3.4. Removal of NXB

The NXB enzyme mix is recommended to remain on the wound bed for at least four hours. Before removal, the persistence of sufficient pain management must be ensured. Afterwards, dressings and the occlusive film can be removed in preferably aseptic conditions. Next, the dissolved eschar has to be sterilely dislodged together with the residuary NXB mix by using instruments such as a spatula or dry sterile gauze. After complete removal, the exposed tissue should be abraded with NaCl-soaked gauze until occurrence of a pinky wound bed with disseminated pinpoint haemorrhages or exposure of a whitish, less vascularized tissue in case of deeper burn injuries. Subsequent to abrasion, chlorhexidine- or polyhexanide-soaked wound dressings are put back on the debrided area for at least another two hours, representing the so-called post-soaking phase.

### 3.5. Evaluation

The visual assessment of wound bed characteristics at this point after completion of enzymatic debridement is crucial for determination of further therapy and, thus, requires special diligence, experience, and documentation. Clinical characteristics such as visible tissues, structures, or bleeding patterns allow for the accurate estimation of burn depth after ED [76]. Similar to classic treatment algorithms, after ED, deeper burns—indicated by persistent paleness and sporadic, large diameter bleedings—should also be followed by skin grafting, whereas more superficial burn patterns—indicated by a pink wound bed or numerous disseminated pinpoint lesions—can undergo a conservative treatment pathway [65].

However, as already known from the initial assessment of burn patterns, post-interventional evaluation is also realised by the respective clinician and thereby contingent on subjectivity and prone to misjudgement. The formation of so-called pseudo-eschar on the surface of the treated area, consisting of fibrin and other wound exudates, starts to complicate sufficient wound bed assessment promptly after completion of ED. This factor makes it nearly impossible to come to a reasonable decision concerning a surgical or conservative treatment regime when wound bed evaluation has not taken place immediately after end of treatment and simultaneously prevents any chance of re-evaluation. Hence, this part of performing ED may represent the most demanding but also crucial point for therapy success.

Since high dependency on a singular evaluation point often poses inconveniences in clinical routine, scientific efforts are being made to implement procedures for better workflow integration and higher interrater reliability. For this reason, the group of Siegwart et al. has tested interrater reliability regarding postinterventional wound bed evaluation and therapy decisions based on short video sequences of 7 to 19 s length [77]. Correspondence of the assessments was significantly dependent on the clinicians’ experience. The conducted survey revealed the highest rates (60%) of consensus in wound bed evaluation in the subgroup with the highest ED experience (>50 ED applications) and the lowest rates (13.3%) in the subgroup with the lowest experience (>10–19 ED applications). The distribution was comparable regarding proposed therapy regime, underlining a high overall interrater variability when evaluating ED. From their results, Siegwart et al. conclude that the consultation of experienced clinicians, as well as detailed media documentation (via photography or video recording) for joint diagnosis and treatment determination, may contribute to more appropriate decision making and thus provide the best patient outcome. Numerous studies have already approved the high correlation of media-supported wound assessment with clinical examination in burns, which is facilitated by generally visual-based evaluation of burn injuries and widespread availability of camera phones [78,79,80,81,82,83]. In this context, the participants of the study have agreed that recording of post-ED results does not only provide a feasible and beneficial tool for wound bed evaluation and therapy determination but also for training purposes and education of less experienced clinicians. Furthermore, digital recording allows a delayed presentation of post-ED results in front of the entire burn team for validation and consensus finding, which also describes the standard at the authors’ clinic.

## 4. Results and Advantages

Since the market approval of NXB, several studies have been published reporting positive and satisfying results in various burn depths, manifesting the additional value of ED in context of initial handling of burn injuries [20,22,66,68,69,84,85,86,87,88,89,90,91]. While initially intended to prevent the requirement of skin grafting, ED is now ordinarily used for general and decent debridement independently of the planned therapeutic regime. Due to its characteristics, majority of studies focus on application on predisposed regions that benefit most from sparing debridement, such as the face, limbs, or genital region [20,21,92]. On the basis of its minimal invasiveness, ED is also appropriate for the successive debridement of different body areas in case of injuries with high TBSA, allowing the stepwise necrectomy of large surfaces without perioperative considerations of classical surgery.

Early studies, now empirically supported by long-term experiences due to an increasing popularity of ED, have already stated several advantages in comparison to classic surgery [69,93,94,95]. While surgery, which is currently the most frequently used method for initial debridement and commonly still represents the SOC, undisputedly offers a sharp and radical excision of damaged structures with—frequently—nothing left but vital tissue on the one hand, it is still highly traumatic, requires OR facilities with anaesthesia, can cause extensive blood loss, and nearly always requires skin grafting on the other hand. On the contrary, ED provides a more selective and aligned way for removal of eschar and, thus, avoids “collateral damage” to surrounding viable tissue, increasing the chances for conservative treatment options by leaving intact dermis with ability for spontaneous reepithelization [25,65,95,96,97]. Eschar removal is perfectly aligned to the vital tissue, precisely revealing the actual burn depth even in complex heterogenous burn patterns or surgical challenging regions. Thereby, the use of ED can minimize blood loss and often obviates the need of skin grafting or, at least, reduces the area requiring surgical excision or transplantation, respectively [95,96]. As ED requires low technological needs, application can be feasible for bed side application depending on localization of burn wounds. This system saves OR capacities on one hand and may further not even require anaesthesiologic assistance due to reported treatment options in local anaesthesia on the other hand [65]. Additionally, feasibility with regional or local instead of general anaesthesia reduces perioperative risk factors. This represents a crucial advantage for patients with chronic obstructive lung disease (COPD) or cardiopulmonary instability [67,98]. Studies have shown that potential renunciation of intubation in patients with COPD, who frequently suffer facial burns due to smoking in combination with home oxygen therapy, can prevent prolonged ventilation time and reduce the length of stay at an intensive care unit [67]. Various studies and case reports, as well as the authors’ experiences have furthermore revealed great capabilities for dissolving circumferential and constrictive eschar, which poses a risk for development of BICS, providing an enzymatic alternative for conventional surgical escharotomy of limbs [86,99,100,101]. The abandonment of pre-soaking appeared feasible in these cases due to the urgent need for action and was not associated with significant restrictions regarding debriding effects. This statement is supported by the authors’ experiences with “fast-track-application”, as discussed previously, which waives the recommended pre-soaking procedure.

In addition to medical aspects, economic issues also play an increasingly important role in today’s hospitals funding. Cost analysis revealed significant savings of EUR 5330 per patient on average when using NXB in comparison to standard treatment, expanding enzymatic debridement also by financial benefits [102].

However, despite various advantages also ED is subject to few limitations: As already implicated by the manufacturer´s guideline, studies have shown the impairment of debridement efficiency due to enzyme inhibition in case of topical pre-treatment with various commonly used agents or ointments. This issue has already been the subject of research at our department aiming for the identification of possibly interfering chemical substances, revealing inhibitory effects for Octenisept^®^ and silver- or copper-bearing agents [75]. Another practical barrier is the reported, lower effectiveness in scalds, leading to higher need of surgical intervention and prolonged hospitalisation [103]. This difficulty may result from premature application of ED prior to actual demarcation of tissue damages, pre-dominantly occurring in scalds or chemical burns and referred to as “burn progression”. Due to this mechanisms, only just vital tissue layers may not be processed in case of early ED but suffer from perishing of pre-damaged dermis later on, once more forming a non-vital wound bed with constrained healing or grafting capabilities. Another occasionally assumed effect traced back to the application of ED is the occurrence of (mild) coagulopathy, which is also reflected by safety notes of drug approval [63,74]. The enzyme bromelain has been associated with coagulation abnormalities in in vitro and in vivo experiments, as well as after oral medication [104,105,106,107]. A case report of Martìn et al. has already drawn attention to this subject in case of topical ED using a bromelain-based approach in burn injuries [108]. When performing ED, it can be recommended to critically evaluate the inclusion of patients with pre-existing risk factors for coagulation abnormalities such as established anticoagulant therapy, low platelet count, or an increased risk of active bleeding (e.g., due to sepsis or gastrointestinal haemorrhage). Significant coagulopathy is supposed to be normalised prior to ED [65]. However, data remains insufficient for final conclusion due to so far poor study design.

## 5. Extended Application Range Based on Expert Consensus

The described advantages in combination with satisfactory results and manifold usability of ED led to the ambition of further standardization of ED-application and identification of potential indications outside market approval. In spite of various national expert councils, the development and update of a European consensus guideline by a panel of 10 or, respectively, 12 international ED-experienced experts in 2017 and 2019 can be considered as most conclusive elaboration concerning use of ED in burn injuries [64,65,109,110]. Various statements were discussed to find general consensus and to provide expert-based handling instructions, resting on until-then conducted scientific studies. The results not only led to confirmation of the manufacturer’s instructions but also to approval of potential exceedance of official market licensing at certain points. In total, consensus was achieved for all discussed statements (68) in 2017 (cut-off for consensus agreement > 50%) and for 42 of 43 discussed statements in 2019 (cut-off for consensus agreement > 80%).

Consenting exceedance of market approval comprised application in paediatric patients [111], utilization for up to 30% TBSA [112], waiver of pre-soaking under certain conditions, applicability for prevention of peripheral BICS, prolonged enzyme application for up to 18 h for improved results, or possible but reluctant retreatment with ED [64,65]. However, explicit surpassing of official recommendations has clearly been pointed out as off-label use. Furthermore, there was also consensus for lower effectiveness in scalds as well as crucial importance of detailed documentation after completion of ED for best treatment determination. Replacement of surgical escharotomy by ED in case of respiratory compromise in circumferential trunk burns was concordantly not recommended, although experts agreed on its preventive potential when applied at early stage [65]. Regarding individual diagnostics, experts agreed on additional value of pre-interventional LDI, which represents the tool of choice in order to identify regions with promising spontaneous healing capabilities or ascertained requirement of skin grafting. In addition, in case of facial application, ophthalmological examination is recommended before and after ED to exclude affection of visual acuity.

Due to the described advantages compared to classic surgery and a progressively expanding field of application possibilities, ED has been successfully implemented in algorithms for initial management of burn injuries in specialised burn centres worldwide. In the author´s clinic, enzymatic treatment represents the first-line therapy for appropriate burn injuries of complex anatomical areas such as hand, face, or genital region.

## 6. Discussion

Surgical as well as enzymatic debridement are established approaches for sufficient eschar removal in burn injuries. In comparison to classic surgery, ED offers various advantages, as described above, and may therefore contribute to a more adequate and convenient treatment of burn injuries for both patients and clinicians. As commonly ED-treated burns in hand, face, or genitals constitute the indication for transferal to a specialised burn centre, ED treatment can regularly be supplied to this group of patients, guaranteeing best patients’ outcome. Studies have reported benefits of ED for high-risk patients with pulmonary disease as well as applicability of ED in patients with dermal illnesses such as systemic sclerosis [67,98,113].

The high convenience of ED for the responsible practitioners is reflected by simplicity regarding determination of ED indication. Although ED is officially recommended to be conducted by an experienced burn surgeon, through its capabilities and characteristics, it appears to be practically also applicable for less qualified clinicians: Whereas post-interventional wound bed evaluation, as outlined previously, surely requires a high degree of experience or even interprofessional exchange for the best decision making, the application of ED does not set high demands on appropriate wound assessment due to its high therapeutic index and suitability for a broad range of burn patterns. In contrast to tangential or epifascial necrectomy, ED does not pose a risk for overtreatment in terms of unnecessarily deep and comprehensive excision after incorrect burn depth assessment by less experienced colleagues. For this reason, the classification as “deep burn”—comprising mixed partial-, deep partial- and full-thickness burns—can be regarded as sufficient categorisation for the implementation of ED. This circumstance may suggest the possibility of implementing ED, after appropriate training, as initial treatment of minor burn injuries also in non-specialised, smaller clinics. Depending on availability of burn centres, it could be a reasonable concept—e.g., when suitable patients seek medical assistance in the evening or during night—to perform photo documentation and analgesia, followed by pre-soaking and ED application before transferring to a specialised clinic the next day where the wound can be appropriately assessed after the inhouse removal of NXB.

The involvement of peripheral clinics through easily applicable approaches for treatment of burn injuries may contribute to improved load control and resource management of specialised burn centres. This suggestion appears reasonable in the context of increasing case numbers at burn centres, whereas the majority of developed countries, including Germany, inconsistently report dropping burn incidences [2,114]. Therefore, it is of special note that increase in case numbers at burn centres appears to be caused by a higher volume of minor burns, limiting capacities, and human resources [2].

Scientific effort is also made regarding the optimal post-ED treatment, comprising the enhancement of wound healing and cicatrisation. Comparison between surgical and enzymatic debridement has already revealed superior scar quality in characteristics such as pigmentation, thickness, relief, and irregularity when performing tissue preserving ED [87]. Measures ensuring the success of scar maturation after ED resemble known strategies including moisturizers, physical therapy, compression garments, silicone, or avoidance of UV exposition [65]. Another scientific approach for improved healing and scarring after ED is the utilization of autologous platelet-rich fibrin (PRF) [115]. Many recent studies have proven that use of PRF can augment re-epithelisation, prevent infection, and, thus, lead to accelerated wound closure [116,117,118,119,120,121]. In a small-scale study of 10 patients with partial to deep burn injuries undergoing NXB treatment, Schulz et al. topically applied PRF on burn wounds achieving satisfactory functional and aesthetic results, even on hard-to-heal wounds [116]. This observation may implicate a practical relevance for inclusion of PRF in advanced ED aftercare; however, the authors consider further research on this object as necessary due to limited number of study participants.

## 7. Conclusions

Taken together, ED represents a strong approach for individualised burn therapy with minimised harm to healthy tissue and, thus, is a powerful device for initial treatment of burn injuries, lower length of hospital stay, and improved patient care by reducing the necessity of surgery, autografting, or blood products. The broad applicability of ED may furthermore serve as an opportunity to also involve non-specialized clinics in the initial treatment of burn injuries. However, the decision of further strategies will still be incumbent upon burn specialists due to experience-based wound bed evaluation after enzymatic intervention.

The reported results in burn injuries have also attracted attention for treatment of other pathologies such as chronic wounds using enzyme-based techniques, which are currently object of ongoing research in this field [122,123,124].

With our article, we illustrate the establishment of ED in initial burn treatment and point to the capability of enzymatic approaches to represent a major pillar in future burn surgery.

## Figures and Tables

**Table 1:** Indications for transport to specialized burn centres.

Transfer to a Specialised Burn Centre Is Mandatory in Case of:
Partial thickness burns of 10% or more body surface area
Full thickness burns of any extent
Burns to hands, face, or genital region
Burns from electricity (incl. lightning)
Chemical burns
Inhalation trauma
Concomitant diseases or injuries predisposing for a complex course of treatment
Requirement of special psychological, psychiatric, or physical care
